# Abnormal Degree Centrality Associated With Cognitive Dysfunctions in Early Bipolar Disorder

**DOI:** 10.3389/fpsyt.2019.00140

**Published:** 2019-03-20

**Authors:** Wenhao Deng, Bin Zhang, Wenjin Zou, Xiaofei Zhang, Xiongchao Cheng, Lijie Guan, Yin Lin, Guohui Lao, Biyu Ye, Xuan Li, Chanjuan Yang, Yuping Ning, Liping Cao

**Affiliations:** ^1^Department of Early Intervention, Affiliated Brain Hospital of Guangzhou Medical University, Guangzhou, China; ^2^Department of Radiology, Affiliated Brain Hospital of Guangzhou Medical University, Guangzhou, China; ^3^Department of Child and Adolescent, Affiliated Brain Hospital of Guangzhou Medical University, Guangzhou, China; ^4^Mental Health Institute, Affiliated Brain Hospital of Guangzhou Medical University, Guangzhou, China

**Keywords:** bipolar disorder, early intervention, functional MRI, degree centrality, cognitive impairment

## Abstract

Delayed diagnosis of bipolar disorder (BD) is common. However, diagnostic validity may be enhanced using reliable neurobiological markers for BD. Degree centrality (DC) is one such potential marker that enables researchers to visualize neuronal network abnormalities in the early stages of some neuropsychiatric disorders. In the present study, we measured resting-state DC abnormalities and cognitive deficits in order to identify early neurobiological markers for BD. We recruited 23 patients with BD who had recently experienced manic episodes (duration of illness <2 years) and 46 matched healthy controls. Our findings indicated that patients with BD exhibited DC abnormalities in frontal areas, temporal areas, the right postcentral gyrus, and the posterior lobe of the cerebellum. Moreover, correlation analysis revealed that psychomotor speed indicators were associated with DC in the superior temporal and inferior temporal gyri, while attention indicators were associated with DC in the inferior temporal gyrus, in patients with early BD. Our findings suggest that DC abnormalities in neural emotion regulation circuits are present in patients with early BD, and that correlations between attention/psychomotor speed deficits and temporal DC abnormalities may represent early markers of BD.

## Introduction

Bipolar disorder (BD) is a severe psychiatric disorder associated with profound mood instability and variability. Due to the risks of psychosis, suicide, chronicity, and recurrence, BD places a tremendous emotional and financial burden on affected patients and their families (1). BD can be divided into type I (BD-I, with mania) or II (recurrent depression with hypomanic phases). BD-I affects ~1.0% of the worldwide population (2) and ~0.1% of the Chinese population (3). Clinicians and researchers have recently suggested that early intervention may reduce this burden, as this strategy has the potential to lessen the severity of the full-blown disorder or delay or even prevent relapse (4). However, initial misdiagnosis and delayed diagnosis of BD are common due to diagnoses based on the appearance of symptoms to date and the overlap of clinical features with other psychiatric disorders (5–7). However, diagnostic validity can be enhanced by adding reliable neurobiological markers for BD.

One challenge in identifying early markers for BD lies in identifying patients in the early stage of the disease. Certain symptoms and impaired functioning may manifest in the prodromal phase, during which the risk of psychosis is substantially increased, indicating that prevention efforts may be justified (8). However, one meta-analysis reported that the sensitivity of current psychopathological criteria for prodromal BD is generally low (9). Recent studies have also suggested that psychiatric problems in young patients may be associated with increased susceptibility caused by neurodevelopmental deficits, and that healthy relatives of patients with psychiatric disorders with high genetic risk are most likely to develop these disorders. Therefore, it may be appropriate to examine these individuals when investigating early biomarkers of such disorders (10). However, the nervous system is still developing in young patients, and the clinical features of BD are atypical in this patient population. Furthermore, healthy relatives of patients are at risk for developing psychiatric disorders other than BD, leading to a lack of consistency in biomarker analyses. Therefore, careful selection of samples for the identification of early biomarkers is critical.

Deficits in sustained attention, verbal memory, and executive functioning are regarded as behavioral markers of BD (11). Moreover, these neurocognitive deficits can be observed even among patients in remission (12, 13), regardless of medications used (14). Interestingly, a meta-analysis revealed that cognitive deficits can be observed in patients experiencing their first BD episode, indicating that cognitive dysfunction is present even in the early stages of disease (15). Furthermore, some studies have suggested that impairments in attention, memory, and executive function can be observed in the prodromal stage, suggesting that cognitive dysfunction can be used as an early marker of BD (16–18).

Imaging studies have hinted at the biological basis of cognitive dysfunction in patients with BD. For example, patients with BD exhibit abnormal activity in the amygdala, frontal cortex, and temporal cortex during cognitive tasks, suggesting that BD is associated with abnormalities in emotion-processing circuitry (10, 19–21). However, the human brain is highly interconnected under normal conditions (22–24), indicating that particular psychological functions are controlled by spatially distributed networks rather than by focal brain areas or structural defects at the tissue level. The degree centrality (DC) approach for analyzing resting state functional MRI (fMRI) data enables researchers to investigate these integrated neuronal networks by measuring the number of instantaneous functional connections (or correlations) between one region and the rest of the brain (25). Notably, such studies have demonstrated that DC abnormalities can be observed in the early stages of several neuropsychiatric disorders, including schizophrenia (26), major depressive disorder (27), and Alzheimer's disease (28). However, it remains to be determined whether such DC abnormalities can be observed in the early stages of BD.

In the present study, we aimed to identify potential neurobiological markers for early BD by comparing resting-state DC between patients with BD and healthy controls. We further aimed to determine whether DC abnormalities are associated with cognitive dysfunction in patients with early BD. To achieve these aims, we recruited patients with BD with a disease duration of <2 years, as the first 2–5 years after illness onset represents an important indicator of clinical and psychosocial deterioration (29). Moreover, this period is thought to represent the critical period for determining long-term outcomes (30, 31). To avoid the confounding effect of different mood states, we selected patients who had recently experienced a manic episode.

## Materials and methods

### Participants

Inpatients and outpatients with BD (*N* = 49) treated at the Affiliated Brain Hospital of Guangzhou Medical University (Guangzhou Huiai Hospital) were included in the study. We also recruited healthy controls matched according to age, gender, and education (*N* = 76). All participants were screened for diagnoses based on the 4th **edition of the Diagnostic and Statistical Manual of Mental Disorders** (DSM-IV) using the Structured Clinical Interview for DSM-IV Axis I Disorders (SCID), which was administered by trained postgraduates or psychiatrists.

Inclusion criteria for the BD group were as follows: (a) diagnosis of BD-I based on DSM-IV criteria, (b) experience of recent manic episode as determined using the SCID, (c) disease duration ≤24 months, (d) right handedness, and (e) Han Chinese ethnicity. Healthy controls (HC) were included if they (a) lacked a lifetime history of Axis I or II psychiatric disorders; (b) had no family history (first- or second-degree relative) of mental disorders; (c) were roughly matched with the BD group according to age, gender, and level of education; (d) were right handed; and (e) were of Han Chinese ethnicity.

Participants were excluded if they had (a) any current Axis I or II diagnosis other than bipolar affective disorder, (b) neurological diseases, (c) a history of disturbance in consciousness after traumatic brain injury, or (d) any mental or physical status that would prevent them from undergoing MRI.

The protocol was carried out in accordance with the Declaration of Helsinki and approved by the Institutional Review Board at the Affiliated Brain Hospital of Guangzhou Medical University (Guangzhou Huiai Hospital). Written informed consent was obtained from all participants prior to enrollment in the study.

### Clinical and Neuropsychological Assessment

Clinical severity was rated using the 17-item Hamilton Rating Scale for Depression (HAMD) and the Bech–Rafaelsen Mania Rating Scale (BRMS) (including two additional items for psychotic symptoms, BRMS-A). Cognitive function was examined in all participants no more than 24 h prior to or after scanning. The test battery included the digit symbol test (DSyT), trail-making test part A (TMT-A), digit span test [(DSpT)-forward (DSpT-F) and -backward (DSpT-B)], and verbal fluency test (VFT) for the assessment of psychomotor speed, attention, working memory, and executive function (15).

### Acquisition of MRI Data

All participants completed a resting-state fMRI scan within 1 day after the interview. During the scan, participants were instructed to relax with their eyes closed but not to fall asleep. Blood oxygenation level-dependent (BOLD) signal were acquired using a 3.0-T Philips Achieva MRI scanner (Philips, the Netherlands). Fast field echo (FFE) echo-planar images (EPI) were acquired using an eight-channel SENSE head coil with the following parameters: repetition time = 2,000 ms; echo time = 30 ms; flip angle = 90°; 33 slices; matrix = 64 × 64; field-of-view = 220 × 220 × 150 mm^3^' voxel size = 3.44 × 3.44 × 4 mm^3^; gap = 0.6 mm; and number of signal averages (NSA) = 1. The resting fMRI scan (8 min, 43 s) comprised 240 contiguous volumes. A T2-weighted anatomical image was also acquired to exclude obvious brain atrophy.

### Preprocessing of MRI Data

MRI data were preprocessed using Statistical Parametric Mapping (SPM8, http://www.fil.ion.ucl.ac.uk/spm), Resting-State fMRI Data Analysis Toolkit (REST) V1.8 (32), and the Data Processing Assistant for Resting-State fMRI Advanced Edition (DPARSFA) V2.3 (33).

The first 10 volumes were removed to ensure signal equilibrium. The remaining data were slice-time corrected for interleaved slice acquisition and three-dimensional head motion. As mentioned above, eight participants were excluded based on six motion parameters with a threshold of 3 mm (or degrees). The remaining data were then spatially normalized to the Montreal Neurological Institute (MNI) space and resampled to 3-mm isotropic voxels. Next, the linear trend of the data was removed, and temporal band-pass filtering (0.01–0.08 Hz) was performed to reduce the effects of low-frequency drift and high-frequency physiological noise. Nuisance signals from white matter and cerebrospinal fluid and the six motion parameters were removed to control for physiological processes and large-scale neural signals. We did not perform spatial smoothing because doing so prior to calculating DC values may introduce artificial local correlations to the adjacent voxels.

### Degree Centrality

Preprocessed data were analyzed by deriving DC measures for every gray matter voxel using the cortical hub analysis procedure (25). For each voxel, we extracted the BOLD time course and correlated it with every other voxel in the brain. For each voxel, *j*, the number of strong voxel-to-voxel correlations (defined as correlation coefficient *r* > 0.25) was computed to determine the DC of *j*. A threshold of 0.25 was chosen to minimize the risk of including voxels whose correlation with the index voxel could be accounted for by noise in the centrality estimate for the index voxel. We computed the binary DC values of the whole-brain network. A map of DC values for each gray matter voxel was obtained for each participant. These maps were then z-transformed to enable group comparisons. Prior to statistical analysis, all individual degree centrality maps were spatially smoothed with a Gaussian smoothing kernel (full-width half maximum, FWHM = 6 mm).

### Statistical Analysis

Analyses of demographic, clinical, and cognitive data were performed using the PASW 18.0 statistical software package (SPSS Inc., Chicago, IL, USA) (34). Student's *t*-tests and χ^2^ tests were used for continuous and categorical variables when appropriate.

Analyses of fMRI data were performed using SPM8. After controlling for the effects of age and gender, two-sample *t*-tests were used to compare DC values between the BD and HC groups. The resulting *t*-maps were corrected for multiple comparisons with the group mask using a Monte Carlo simulation (AlphaSim by B. Douglas Ward) in REST at a corrected *P* < 0.05 (cluster connection radius = 4 mm, 5,000 simulations, FWHM = 6 mm, voxel *P* = 0.001 and cluster size > 12 voxels).

Clusters in which DC values differed significantly between the BD and HC groups were treated as regions of interest (ROIs). The DC values of voxels in the ROIs were averaged to yield DC values for each ROI, which were extracted using SPM8.

While controlling for the effects of age and gender, partial correlation analyses (PASW 18.0) were performed to examine associations between DC values for each ROI and cognitive data (e.g., DSyT, DSpT-F, DspT-B, VFT, and TMT-A results) in patients and controls. The Bonferroni method was used to correct for multiple comparisons. Since there were five cognitive components and DC abnormalities in six brain regions, *P*-values of 0.0017 (0.05/30) were considered statistically significant. Linear regression analyses were used to further assess correlations between DC and cognitive function in patients with early BD. Pearson's correlation analyses were used to examine correlations between DC values and demographic (i.e., age and years of education) and clinical data (i.e., age at onset, duration of illness, number of episodes, and HAMD and BRMS [including BRMS-A) scores] in the BD group. Since there were two demographic variables, six clinical variables, and six ROIs, *P*-values of 0.0010 (0.05/48) and 0.0042 (0.05/12) were considered statistically significant in the BD and HC groups, respectively.

## Results

### Early BD Is Associated With Cognitive Deficits

To exclude the potential effects of confounding factors, we discarded data for four patients with obvious brain atrophy, four patients with comorbid anxiety disorders, two patients with a diagnosis of BD type II, two patients with an illness duration >24 months, and one patient with a minority ethnicity. We also discarded data for six patients and two HCs with excessive head motion (see fMRI Data Preprocessing). Following these exclusions, data from 23 patients with BD and 46 HCs were suitable for further analyses. Overall, there were no significant differences in age, gender, or education level between the BD and HC groups (Table 1).

**Table 1:** Demographic, clinical, and cognitive data for patients with early BD and HCs.

	**BD (*n* = 23) Mean ± SD**	**HC (*n* = 46) Mean ± SD**	***T/χ^2^***	***P***
**DEMOGRAPHIC DATA**				
Age (years)	23.61 ± 4.93	23.79 ± 3.16	0.160	0.874
Gender (Male/Female)	14/9	28/18	0.000	1.000
Education (years)	12.17 ± 3.33	12.96 ± 2.38	1.007	0.321
**CLINICAL DATA**				
Age at onset (years)	22.83 ± 4.84	–	–	–
Illness duration (months)	11.17 ± 9.46	–	–	–
Number of episodes	1.91 ± 1.00	–	–	–
Psychotic symptoms, *n* (%)	16 (69.57)	–	–	–
Family history, *n* (%)	9 (39.13)	–	–	–
HAMD score	4.96 ± 4.45	–	–	–
BRMS score	9.65 ± 7.01	–	–	–
BRMS-A score	1.17 ± 2.71	–	–	–
Medication, n (%)				
Lithium or Valproate	22(95.7)	–	–	–
Antipsychotics	23(100.0)	–	–	–
Antidepressant	3(13.0)	–	–	–
Benzodiazepine	12(52.2)	–	–	–
**COGNITIVE DATE**				
**Psychomotor Speed**				
DSyT score^**^	38.261 ± 13.16	68.37 ± 11.49	9.772	0.000
TMT-A time (seconds)^**^	61.69 ± 28.53	30.84 ± 6.53	−5.117	0.000
**Attention**				
DSpT-F score	8.043 ± 1.52	8.48 ± 1.60	1.080	0.284
**Working memory**				
DSpT-B score^*^	5.17 ± 1.61	6.20 ± 1.73	2.360	0.021
**Executive function**				
VFT score	17.17 ± 4.52	19.54 ± 5.18	1.866	0.066

*BD, bipolar disorder; HC, healthy control; SD, standard deviation; HAMD, Hamilton Rating Scale for Depression; BRMS, Bech-Rafaelsen Mania Rating Scale; BRMS-A, BRMS-additional items for psychotic symptoms; DSyT, digit symbol test; TMT-A, trail making test part A; DSpT-F, digit span test-forward; DSpT-B, digit span test-backward; VFT, verbal fluency test*.

* P < 0.05;

** *P < 0.01*.

Our findings indicated that patients with early BD performed worse than HCs on almost all cognitive domains assessed, including psychomotor speed, attention, working memory, and executive function. However, differences in DSpT-F and VFT scores between the groups did not reach statistical significance (*P* > 0.05) (Table 1).

### DC Abnormalities Are Present in Patients With Early BD

**Figure 1:**
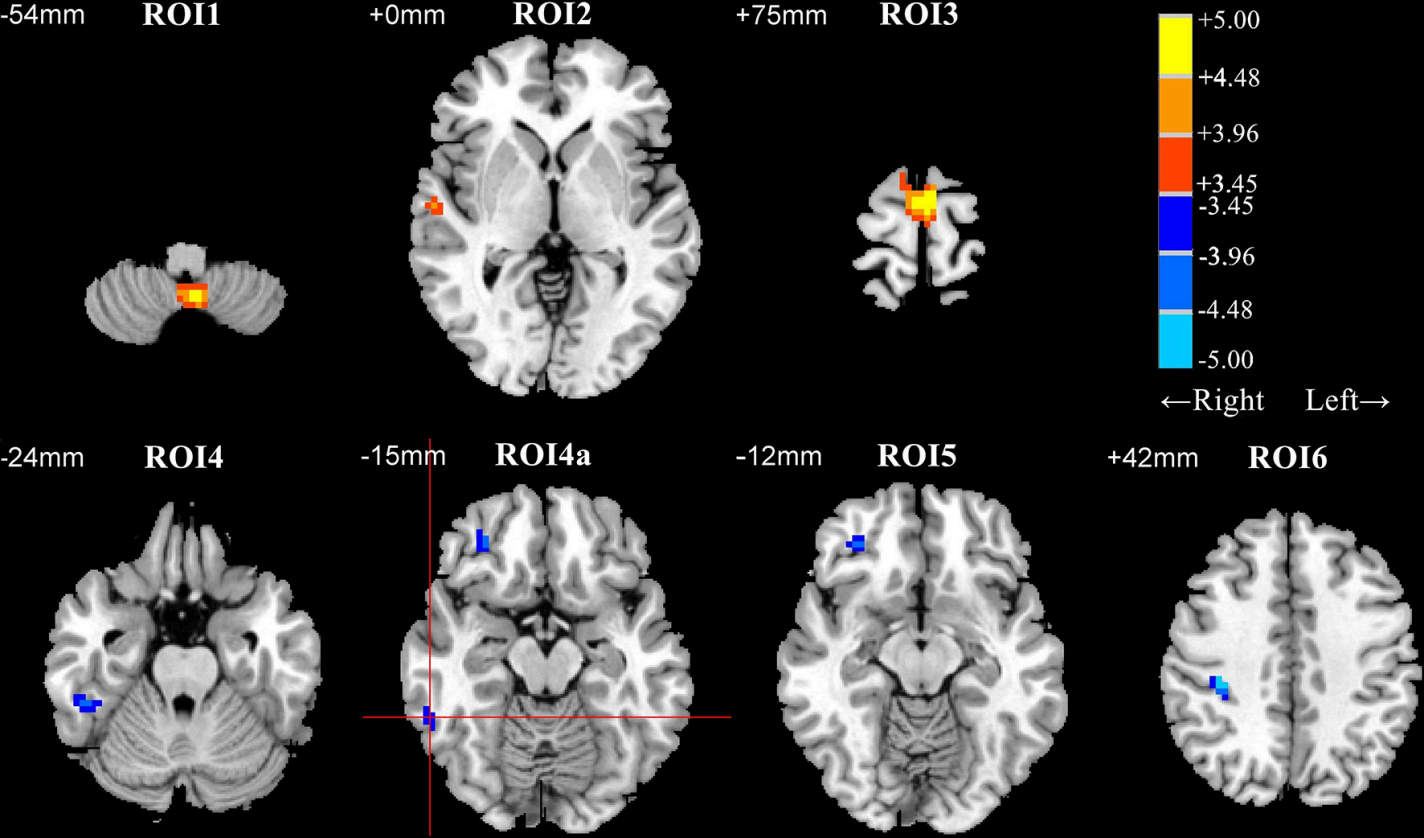
Differences in degree centrality (DC) between patients with early bipolar disorder (BD) and healthy controls (HCs) in the resting state (group analysis, two-sample *t*-test, AlphaSim correction (cluster connection radius = 4 mm, 5,000 simulations, FWHM = 6 mm, voxel *P* = 0.001, and cluster size ≥ 12 voxels). ROI1, posterior lobe of the cerebellum; ROI2, right superior temporal gyrus; ROI3, supplementary motor area; ROI4 and ROI4a, right inferior temporal gyrus; ROI5, right orbital part of the middle frontal gyrus; ROI6, right precentral gyrus and postcentral gyrus. Regions with decreased and increased DC values in patients with BD are shown in warm and cold colors, respectively. The red cross distinguishes ROI4a from other clusters. ROI, region of interest; FWHM, Full-width at half-maximum.

Patients with early BD exhibited DC abnormalities mostly in the frontal and temporal areas. Specifically, when compared to HCs, patients with BD exhibited significant DC decreases in the posterior lobe of the cerebellum (ROI1); the right superior temporal gyrus (ROI2); and the bilateral superior frontal gyrus, medial frontal gyrus, and supplementary motor area (ROI3). Furthermore, when compared to HCs, patients with BD exhibited significant DC increases in the right inferior temporal gyrus (ROI4 and ROI4a), the right orbital part of the middle frontal gyrus (ROI5), and the right precentral and postcentral gyri (ROI6) (Table 2 and Figure 1).

**Table 2:** Degree centrality differences between patients with early BD and HCs during the resting state.

**Comparison**	**MNI Coordinates**			***T***	**Voxels**	**ROI**
**Brain region**	**X**	**Y**	**Z**			
**BD<HC**						
Posterior Lobe of the Cerebellum^*^	−6	−57	−54	4.663	66	1
Right Superior Temporal Gyrus^*^	60	−12	0	4.205	18	2
Bilateral Superior Frontal Gyrus, Medial Frontal Gyrus, and Supplementary Motor Area^*^	−3	−12	75	5.445	76	3
**BD>HC**						
Right Inferior Temporal Gyrus^*^	51	−39	−24	−4.426	20	4
Right Inferior Temporal Gyrus^*^	57	−45	−15	−3.782	12	4a
Right Orbital Part of the Middle Frontal Gyrus^*^	33	39	−12	−4.415	19	5
Right Precentral Gyrus and Postcentral Gyrus^*^	33	−30	42	−4.923	60	6

*BD, bipolar disorder; HC, healthy control; MNI, Montreal Neurological Institute space; ROI, region of interest for correlation analyses; FWHM, full-width at half-maximum*.

* *Regions showing significant differences after AlphaSim correction (cluster connection radius = 4 mm, 5,000 simulations, FWHM = 6 mm, voxel P = 0.001, and cluster size ≥12 voxels)*.

### DC Abnormalities in the Temporal Lobe Are Correlated With Psychomotor Speed and Attention in Patients With Early BD

**Figure 2:**
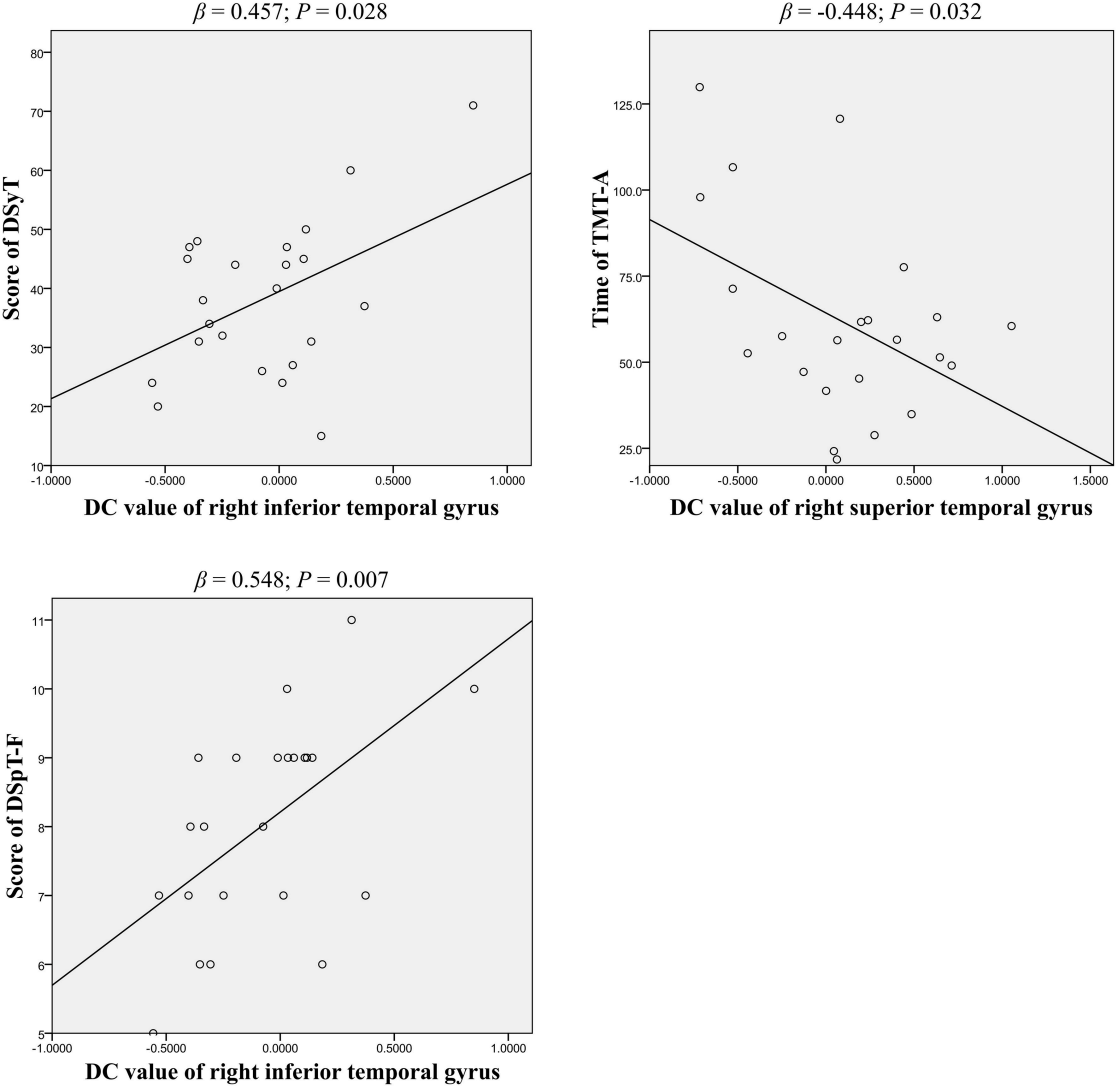
Linear regression of significant correlations between DC and cognitive function in patients with early BD (curve estimation, linear model). DSyT, digit symbol test; TMT-A, trail making test part A; DSpT-F, digit span test-forward; DC, degree centrality; BD, bipolar disorder.

Partial correlation analyses were used to assess the association between DC patterns and cognitive function (Table 3). Our findings indicated that results on two tests of psychomotor speed, the DSyT and TMT-A, were correlated with DC values in the right inferior (*R* = 0.462, *P* = 0.035) and superior (*R* = −0.497, *P* = 0.022) temporal gyri in patients with BD. There was also a correlation between DC values in the right inferior temporal gyrus and scores on the DspT-F (*R* = 0.541, *P* = 0.011) in patients with BD. Linear regression analyses indicated that DC values in the right inferior temporal gyrus exhibited a positive association with scores on the DSyT (β = 0.457, *P* = 0.028) and DSyT-F (β = 0.548, *P* = 0.007), and that DC values in the right superior temporal gyrus exhibited a negative association with time on the TMT-A (Figure 2).

**Table 3:** Partial correlations between DC in six ROIs and cognitive function in patients with early BD and HCs.

**Group**	**Psychomotor Speed**		**Attention**	**Working memory**	**Executive function**
**ROI**	**DSyT**	**TMT-A**	**DSpT-F**	**DSpT-B**	**VFT**
**BD (*****n*** **=** **23)**					
1	0.474^*^	−0.339	0.366	0.343	0.374
2	0.114	−0.497^*^	−0.256	0.080	0.195
3	−0.190	−0.068	−0.137	−0.363	0.021
4	0.462^*^	0.033	0.541^*^	0.296	−0.195
5	−0.270	0.020	−0.242	−0.273	−0.316
6	−0.087	0.323	0.315	0.385	0.039
**HC (*****n*** **=** **46)**					
1	0.348^*^	−0.284	−0.024	0.045	−0.204
2	−0.212	0.081	0.082	−0.087	−0.021
3	−0.012	0.129	−0.064	0.173	0.285
4	−0.091	−0.010	0.053	−0.069	0.178
5	−0.063	−0.044	−0.063	−0.336^*^	−0.214
6	0.002	−0.054	−0.110	0.052	0.008

*ROI, region of interest; BD, bipolar disorder; HC, healthy control; DSyT, digit symbol test; TMT-A, trail making test part A; DSpT-F, digit span test-forward; DSpT-B, digit span test-backward; VFT, verbal fluency test*.

* *P < 0.05*.

In HCs only, we observed a correlation between DC values in the right orbital part of the middle frontal gyrus and scores on the DSpT-B (*R* = −0.336, *P* = 0.026). DC values in the posterior lobe of the cerebellum were associated with scores on the DSyT, although this correlation was similar in both patients with BD (*R* = 0.474, *P* = 0.030) and HCs (*R* = 0.348, *P* = 0.021). However, these correlation results did not survive Bonferroni correction (*P* < 0.0017).

### Correlations Between DC Values for ROIs and Demographic/Clinical Data as Potential Markers for Early BD

Pearson's correlation analyses were used to examine correlations between DC values for ROIs and demographic/clinical data (Table 4). We observed correlations between DC values in the bilateral superior frontal gyrus, medial frontal gyrus, supplementary motor area, and age in patients with BD (*R* = 0.424, *P* = 0.044). In contrast, negative correlations were observed between DC values in the right orbital part of the middle frontal gyrus and age in HCs (R = −0.379, *P* = 0.009), but not in patients with early BD. Interestingly, we further observed an association between DC values in the posterior lobe of the cerebellum and the duration of illness (*R* = 0.443, *P* = 0.034) in patients with BD. Finally, we observed a correlation between DC values in the right superior temporal gyrus and HAMD scores (*R* = −0.495, *P* = 0.016) in patients with BD. Interestingly, DC values in the right inferior temporal gyrus were also correlated with HAMD scores in patients with BD, but only at a marginal level (*R* = 0.413, *P* = 0.050).

**Table 4:** Correlations between DC in six ROIs and demographic and clinical data in patients with early BD and HCs.

**Group**	**Age**	**Education**	**Age at onset**	**Illness duration**	**Number of episodes**	**HAMD**	**BRMS**	**BRMS-A**
**ROI**								
**BD (*****n*** **=** **23)**								
1	0.031	0.342	−0.023	0.443^*^	0.392	0.063	0.240	−0.184
2	0.261	0.057	0.339	−0.359	−0.163	−0.495^*^	−0.288	−0.030
3	0.424^*^	0.149	0.317	0.275	−0.037	0.152	0.052	−0.189
4	−0.208	0.219	−0.220	0.170	0.129	0.413	0.161	0.124
5	0.238	−0.099	0.190	0.010	−0.180	0.126	−0.043	0.029
6	0.045	0.324	0.106	−0.124	−0.126	0.178	0.018	−0.211
**HC (*****n*** **=** **46)**								
1	−0.130	0.055	–	–	–	–	–	–
2	0.164	−0.248	–	–	–	–	–	–
3	0.185	−0.041	–	–	–	–	–	–
4	0.069	0.021	–	–	–	–	–	–
5	−0.379^**^	−0.006	–	–	–	–	–	–
6	0.045	0.186	–	–	–	–	–	–

*ROI, region of interest; BD, bipolar disorder; HC, healthy control; HAMD, Hamilton Rating Scale for Depression; BRMS, Bech-Rafaelsen Mania Rating Scale; BRMS-A, BRMS-additional items for psychotic symptoms*.

* P < 0.05;

** *P < 0.01*.

## Discussion

In the current study, we performed a resting-state fMRI analysis of DC throughout the whole brain to investigate potential markers of early BD. Our findings showed that (a) early BD was associated with cognitive deficits, and that (b) patients with BD exhibited DC abnormalities primarily in frontal and temporal areas when compared with HCs, and (c) the correlations between temporal DC values and both psychomotor speed and attention were specific to patients with early BD. (d) We also observed correlations between age and DC values in the bilateral superior frontal gyrus, medial frontal gyrus, supplementary motor area, and between the duration of illness and DC values in the posterior lobe of the cerebellum in patients with early BD. Taken together, these results indicate that brain imaging and cognitive testing may yield potential markers of early BD in patients with a disease duration ≤2 years. Identifying early markers of BD will not only aid in the clinical diagnosis of the disorder, but will also help to promote recovery via early therapeutic intervention.

In the current study, we found DC abnormalities in patients with early BD occurred mostly in frontal and temporal areas, which is consistent with previous study. Palaniyappan et al. also used resting-state DC methods and found abnormalities in the bilateral precentral gyrus and inferior temporal gyrus in patients with BD experiencing psychosis (35). However, in contrast to previous study, DC abnormalities were found only unilateral in our study. The possible reason is only patients with a disease duration ≤2 years included and not all of them with psychotic symptoms in the current study, which needs further exploration.

We observed that decreased DC in the bilateral superior frontal regions was positively associated with age among patients with BD, whereas decreased DC in the right orbital part of the middle frontal gyrus was negatively associated with age in HCs. These findings indicate that younger patients exhibited more severe decreases in DC in the superior frontal regions, and that they did not experience the DC suppression in the orbitofrontal cortex that occurs during maturation. Adler and colleagues reported significantly decreased fractional anisotropy (FA) in superior frontal white matter tracts in young patients with first-episode BD (36), which presented that altered connectivity between superior frontal regions and the rest of the brain in young BD patients. So, young patients with BD may exhibit neurodevelopmental abnormalities partly in frontal brain regions. The other brain regions abnormally correlated with frontal need to be further confirmed with a seed-based functional connectivity. In the present study, we observed decreases and increases in DC in the superior temporal gyrus and inferior temporal gyrus in patients with early BD, respectively. Consistently, previous studies have reported increases and decreases in gray matter volume in the superior temporal gyrus (37) and inferior temporal gyrus (38), respectively, in patients with first-episode BD. Such studies have suggested that these differences are determined by neurodevelopmental differences in gray matter volume that precede symptom onset (38). Previous studies have also reported that young patients with BD and those at risk for BD exhibit altered resting-state fMRI activity in frontotemporal circuits (39) and large-scale networks (40), abnormally decreased prefrontal cortical-amygdala functional connectivity during tasks (41–43), abnormally decreased orbitofrontal cortex and anterior cingulated cortex gray matter volume (43), and abnormally reduced FA in white matter tracts connecting prefrontal and subcortical regions (44–46). The frontal and temporal cortices are regarded as important components of the emotion-regulation circuitry (47), and alterations in these regions may be associated with characteristic symptoms of BD such as emotional over-reactivity and emotional dysregulation (10, 48). As previously mentioned, examining young patients with BD or those at risk for BD may help to identify early markers of BD. A recent review reported that individuals with BD exhibit increased activation in the superior frontal gyrus and medial frontal gyrus when compared with healthy controls, independent of the fMRI task used, supporting the notion that such activation can be used to predict the risk of BD (49). Taken together, the accumulated evidence suggests that abnormalities in emotion-regulation circuits are present in early BD.

In the present study, we observed correlations between DC abnormalities and neurocognitive dysfunction in the patient group, which may represent additional markers for early BD. Interestingly, results for two different tests of psychomotor speed, DSyT and TMT-A, were correlated with DC values in the right inferior and superior temporal gyri in patients with early BD, suggesting that DC abnormalities in the temporal lobe are associated with deficits in psychomotor speed in early BD. Indeed, among all neurocognitive comparisons, the largest difference between patients in the early stage of BD and HCs was observed with regard to psychomotor speed. This finding has previously been documented in the literature (50, 51) and has also been observed in healthy family members (52, 53), suggesting a potential early marker for BD. One study of cerebral blood flow in BD revealed a correlation between poor performance on the TMT and greater perfusion in the anterior temporal region using single-photon emission computed tomography (54). Hartberg et al. also reported that the positive relationship between inferior temporal surface area and digit symbol test scores is specific to BD (55). Surface area is proposed to be mainly determined during embryonic and neonatal life, without undergoing major subsequent changes (56), and thus may provide a window for investigating early neurodevelopmental disturbances. Consistent with the findings of previous studies, our results confirm the relationship between psychomotor speed and the temporal lobe in patients with BD, which may reflect in part the biological mechanisms underlying neurocognitive dysfunction and provide a marker for the early identification of BD.

We also observed a positive correlation between DSpT-F scores and DC values in the superior temporal gyrus in patients with early BD. Although DSpT-F performance was similar between the two groups, likely due to the insufficient difficulty of the test, previous studies have reported early BD is associated with impairments in attention (18, 50). In addition, our results indicate that changes in brain function may be involved in the potential neurobiological basis of changes in cognitive performance, further supporting the notion that assessments of cognitive function can be combined with brain imaging analyses to improve early identification of BD. Similarly, Ozerdem et al. reported that patients with BD exhibit disturbances in functional long-range connectivity between the frontal and temporal lobes, and between temporal and parietal brain regions, during a cognitive paradigm requiring attention and immediate recall (57). These findings further suggest that temporal abnormalities are associated with impairments in attention in patients with BD. Frazier and colleagues also reported that early-onset BD is associated with deficits in temporal cortical gray matter, which is involved in the control of attention (58).

Attention and psychomotor speed are fundamental components of the cognitive system (59), and performance in both domains is correlated (60). Therefore, they are often studied together. In the present study, DC values related to attention and psychomotor speed performance were similar in the temporal lobe in patients with BD. A recent study reported that attention/psychomotor speed could prospectively predict social impairment 18 years later in patients with mood disorders (61). Therefore, the correlation between attention/psychomotor speed deficits and temporal DC abnormalities may provide insight into the etiology of BD and enable the identification of clinical targets for early treatment. Although we observed that DC abnormalities were associated with neurocognitive dysfunction in early BD, these results did not survive Bonferroni correction. Therefore, these results may represent false positives and should be interpreted with caution.

The present study possesses some limitations of note, which should be considered when interpreting our findings. First, our sample size was relatively small, particularly in the BD group, which may have limited statistical efficiency. Second, the results of this study are somewhat limited by variations in the medication status of the BD group, although there is no evidence that these medications exert differential effects on measures of centrality. However, volumetric measures may be affected by both antipsychotics and lithium (62). Third, our study is limited by selection bias in the BD group, which only included patients who had recently experienced a manic episode.

To the best of our knowledge, the present study is the first to investigate the associations between resting-state network abnormalities and cognitive function in patients with early-stage BD. Our findings suggest that early BD involves DC abnormalities in emotion regulation circuits, which are correlated with deficits in attention/psychomotor speed. Such Future longitudinal studies should include larger samples of patients with BD in different mood states, as well as individuals at risk for BD, in order to more fully explore the potential of abnormal brain network properties as early markers of BD.

## Author Contributions

LC, YN, and CY designed the project. WD, BZ, WZ, XZ, XC, LG, and YL performed the experiment. WD, BZ, WZ, XZ, GL, BY, and XL analyzed the data. WD, WZ, and LC wrote the manuscript. All authors have read and approved the final version of the manuscript.

### Conflict of Interest Statement

The authors declare that the research was conducted in the absence of any commercial or financial relationships that could be construed as a potential conflict of interest.
